# Trends of caesarean section deliveries in Pakistan: secondary data analysis from Demographic and Health Surveys, 1990–2018

**DOI:** 10.1186/s12884-020-03457-y

**Published:** 2020-12-02

**Authors:** Aaisha Amjad, Abeeha Imran, Nabeeha Shahram, Rubeena Zakar, Ahmed Usman, Muhammad Zakria Zakar, Florian Fischer

**Affiliations:** 1 Institute of Social and Cultural Studies, University of the Punjab, Lahore, Pakistan; 2 Department of Public Health, Institute of Social and Cultural Studies, University of the Punjab, Lahore, Pakistan; 3 University of Okara, Okara, Pakistan; 4 Institute of Public Health, Charité – Universitätsmedizin Berlin, Berlin, Germany; 5 Institute of Gerontological Health Services and Nursing Research, Ravensburg-Weingarten University of Applied Sciences, Weingarten, Germany

**Keywords:** Caesarean delivery, Pregnancy complication, Antenatal care visit, Vaginal birth

## Abstract

**Background:**

Pakistan is among those countries where the number of caesarean section births has increased unusually in the past two decades. Therefore, the aim of the present study is to analyse the trend of caesarean section deliveries among child-bearing women (aged 15–49 years) in Pakistan and to identify maternal socio-demographic factors and pregnancy-related variables associated with the change in caesarean deliveries from 1990 to 2018.

**Methods:**

Secondary data from Pakistan Demographic and Health Surveys (1990–2018) were analysed. The analysis of data was confined to child-bearing mothers. Sample sizes were 4029, 5721, 7461 and 8287 for the time periods of 1990–91, 2006–07, 2012–13 and 2017–18, respectively. Socio-demographic information of the mothers and pregnancy-related variables were taken as independent variables for the present study. The association between independent variables and caesarean deliveries was measured in terms of unadjusted odds ratios (OR) and adjusted OR (AOR).

**Results:**

The percentages of the mothers who had at least one delivery during the 5 years prior to each survey who had caesarean deliveries increased continuously from 3.2% in 1990–91 to 19.6% in 2017–18. Results indicate that mothers over 24 years of age, located in Punjab, from the richest socio-economic class and living in urban areas were more likely to have delivered by caesarean section. Mothers with a first child in birth order and who had five and more children, as well as mothers who had more antenatal care visits and delivered babies in private hospitals showed a higher probability of caesarean section births.

**Conclusions:**

The findings of the present study confirm the gradual upsurge in the percentage of mothers delivering by caesarean section during the past two decades in Pakistan. Against this backdrop, some measures need to be taken by health departments to regulate the number of caesarean deliveries. Awareness among women about pregnancy complications and elaborated details by gynaecologists about the medically indicated reasons for caesarean delivery are a few important steps in Pakistan that can help in reducing caesarean deliveries which are not medically indicated.

## Background

Convenient and immediate provision of health care facilities for pregnant women in all countries across the globe is highly important [1]. The instant availability of obstetric care for pregnant women also includes the provision of medical facilities for caesarean section deliveries [2]. Although caesarean sections are considered to be a safe surgical procedure [3], the progressive increase in caesarean births during the past few years has attracted the attention of public health experts globally [4]. In 2018, more than half of all deliveries were caesarean sections in Brazil, Egypt and Turkey. Data from the United States of America, Australia and Germany reveal that almost one in every three pregnant women has a caesarean section delivery [5]. Similarly, a rising trend of caesarean section deliveries has been documented in South Asian countries including Pakistan, where it increased from 3.2% (1990) to 20% (2018) [5, 6].

From 1985 to 2015, the international healthcare community considered the ideal rate for caesarean sections to be between 10 and 15%. However, since the large increase in caesarean section rates, the World Health Organization no longer recommends a specific rate for countries to achieve related to their population level [7]. The findings of some ecological studies reveal that caesarean deliveries do not tend to reduce the mortality rate when it is above 10%. Rather, the chances of mother and foetus mortality increases when the caesarean rate exceeds 15% [8–10]. Previous research highlights that the mortality rate becomes 2 to 4 times higher among the women who delivered through caesarean section compared to those who had a vaginal delivery [8, 9].

Although the new surgical techniques have played a significant role in reducing the postoperative implications, the persistent risks related to blood loss during operation, injury to baby, infection of the uterus scar and prolonged hospital stay cannot be completely overruled [10]. Furthermore, many studies reveal that women who deliver through caesarean section face adverse emotional and psychological problems, including postpartum depression and a saddening experience of childbirth after delivery [8–10]. In addition, research also shows that babies who are born through caesarean section may have a higher probability of experiencing weak immunity and respiratory issues in their later life [11].

Dystocia, previous delivery through caesarean section, size of the baby, cephalopelvic disproportion, prolonged labour and multiple gestations are the most frequent medical indications behind caesarean deliveries [12]. However, the request of the mother for caesarean section can also be considered as a non-medical reason that has contributed to the upsurge of the caesarean rate [13, 14]. Literature also shows that mothers have several cultural or personal reasons behind requesting a caesarean delivery that may include their previous delivery experience, fear of vaginal birth and prolonged labour, and the cultural acceptability of caesarean sections [13]. Nevertheless, in Pakistan, a doctor’s referral to perform caesarean section is considered to be a dominating reason over the pregnant woman’s choice [4]. The increasing rate of caesarean deliveries in Pakistan is somewhat astonishing as most of the mothers residing in the country deliver babies at home. However, keeping in mind the misuse of caesarean sections, it can be speculated that gynaecologists may perform non-medically inflicted caesarean surgery to gain financial benefits, for time convenience and to gain surgical experience [4].

The discussion mentioned above highlights medical and non-medical factors contributing to an increase in the caesarean section rate in several countries around the globe. Due to the unusual upsurge in the caesarean rate during the last two decades, it is highly important to focus on actual data and time trends informing about the factors associated with caesarean deliveries. A previous study investigated these factors in Pakistan based on data from the Pakistan Demographic and Health Survey (PDHS) 2012–13 [3]. Another study was based on the previous three PDHS waves (1990–91, 2006–07 and 2012–13) but included only socio-demographic characteristics and not pregnancy-related variables [15]. The aim of this study is to fill the existing knowledge gap. The purpose of our study is 1) to analyse the trend of caesarean deliveries among child-bearing women in Pakistan and 2) identify various maternal socio-demographics and pregnancy-related variables associated with the change in caesarean deliveries over time from 1990 to 2018.

## Methods

### Sample

Secondary data from four waves (1990–2018) of PDHS were used for the present study. Demographic and Health Surveys (DHS) are conducted worldwide with the aim of providing consistent and authentic information about fertility and reproduction, maternal and childhood morbidity, the mother’s nutritional level and domestic violence. The DHS datasets used the same stratified random sampling technique to select the participants for the research. The present study used the datasets of PDHS conducted in 1990–91, 2006–07, 2012–13 and 2017–18. Trained interviewers were hired to conduct surveys; they obtained the information required from the subjects through filling out the systematically designed questionnaire. The representative samples of ever-married women were 6611 (1990–91), 10,023 (2006–07), 13,558 (2012–13) and 15,068 (2017–18). The overall response rate was found to be above 90% for the data obtained during all four waves of PDHS. In the present study, the research data analysis was limited to mothers aged 15–49 years who had had at least one delivery in the 5 years prior to each survey. Therefore, the sample was 4029 in 1990–91, 5721 in 2006–07, 7461 in 2012–13 and 8287 in 2017–18.

### Variables

We used caesarean section delivery as the outcome variable for the present study. Mothers were asked about the mode of their last delivery in the 5 years prior to each PDHS. The caesarean birth was labelled as “delivery by caesarean section” in the four sets of PDHSs, although there is no information about medical or non-medical reasons for carrying out caesarean surgery.

Independent variables included socio-demographic characteristics of mothers and variables related to the pregnancy (e.g. pregnancy complications, antenatal care visits, termination of pregnancy, place of delivery).

Maternal socio-demographic variables analysed in the study are comparable to a previous study [4]. It included the mother’s age at delivery of child (categorized to allow for large enough sub-groups: “< 18”, “18–20”, “21–24” and “> 24 years”), level of education (“no education”, “primary education”, “middle education”, “secondary education” and “higher education”), employment status (unskilled, skilled, technical/professional/managerial post, not employed), wealth index in quintiles (“poorest”, “poorer”, “middle”, “richer” and “richest”), place of residence (“urban” and “rural”) and regional areas (“Islamabad Capital Territory Punjab”, “Balochistan”, “Sindh”, “Khyber Pukhtunkhwa”, “Gilgit Baltistan”, “Azad Jammu and Kashmir” [AJK] and “FATA”). The earlier surveys of the PDHS do not provide data about Gilgit Baltistan as it was not recognised as a province before 2012. Moreover, Azad Jammu and Kashmir and FATA were only included in the most recent PDHS data conducted in 2017–18.

The birth order variable used in the study indicates the order of a child born to a mother during the previous 5 years and was recoded as “1”, “2–3”, “4–5” and “6 and above”. Pregnancy-related health concerns were measured by variables that included pregnancy complication (“yes” and “no”) and the number of antenatal care visits during pregnancy, that was recoded as (“none”, “1–3” or “4 and above”). The response of mothers was termed “yes” for those who were informed about any health problem, such as high blood pressure, diabetes or obesity, during their visit to a gynaecologist. Additionally, the place of delivery for a baby was recoded as “private” and “public” set-ups that included the private hospitals/clinics and government hospitals, respectively. We excluded all those deliveries that took place at home because delivering babies through caesarean section is not possible at home. The number of children born to a women during 5 years prior to each PDHS were recoded in the categories “1–2”, “3–4” and “5 and above”. Mothers were asked questions regarding whether they had ever had a terminated pregnancy or not and the response categories were divided into “yes” and “no”.

The characteristics of the newborn included the size and weight of the baby at birth. Information on birth weight was acquired from the baby’s birth card. If the birth card was not available, the mother was asked about the weight of the baby at birth. The variable was dichotomised as “below average” (< 2500 g) and “normal” (≥ 2500 g). Fewer than 2% of mothers were able to relate the weight of the baby at birth in all PDHS. Therefore, they provided information about the size of the newborn at the time of delivery. The variable presenting the size of the baby was also recoded as “normal” and “below average”. Recoded variables related to the size and weight of the baby were combined together to obtain a new variable related to the size of the baby at birth that was considered as “normal” and “below average”.

### Data analysis

Data were analysed by using SPSS version 21. We used the sample weights provided by the DHS to deal with the complex sampling design used in the surveys. The weighting enables us to generalise the findings of the present study to all women of the retrospective age group living in Pakistan. Descriptive statistics were shown in the form of frequencies and percentages. A simple binary and multivariate logistic regression were used to assess the association between the independent variables and the dependent variable. Multicollinearity was tested by using the variance inflation factor. Its results indicated no multicollinearity, therefore, all variables could be retained in the model. The association between a dependent variable (caesarean births) and independent variables was indicated by the values of OR and AOR with 95% confidence intervals (CI). The inclusion of independent variables in the multivariate logistic regression analysis was based on the significance level of *p < 0.05.* All the independent variables (*p < 0.05* in binary logistic regression) were entered simultaneously to calculate the AOR through multivariate logistic regression analysis.

## Results

### Maternal socio-demographic characteristics

Of the mothers who had a delivery in the 5 years preceding the survey, 40.5% were less than 18 years old in 1990–91. This percentage reduced slightly in 2006–07 (39.6%) and 2012–13 (27.2%). The trend continued in 2017–18 and the percentage was further reduced to 26.8%. Regarding education, 76.7% women were uneducated in 1990–91. However, this trend reduced in 2006–07 (66.6%), 2012–13 (55.8%) and 2017–18 (50.4%). The majority of mothers were non-working in 1990–91 (85.0%), which decreased by a small proportion in 2006–07 (71.5%) and 2012–13 (72.23%). The percentage of non-working mothers was highest in 2017–18 (86.7%). Additionally, the proportion of women living in rural areas and residing in the Punjab region expanded over the years (Table 1). The percentages of mothers who had caesarean section was almost 3.2% in 1990–91, 7.8% in 2006–07, 13.6% in 2012–13 and 19.6% in 2017–18. The distribution of caesarean sections among socio-demographic and pregnancy-related characteristics is shown in Table 2.

**Table 1:** Socio-demographic characteristics of child-bearing women aged 15–49 years (weighted percentages)

	PDHS 1990–91 (***n*** = 4029)		PDHS 2006–07 (***n*** = 5721)		PDHS 2012–13 (***n*** = 7461)		PDHS 2017–18 (***n*** = 8287)	
	n	%	n	%	n	%	n	%
**Mother’s age at birth (in years)**								
Less than 18	1631	40.5	1902	39.6	2025	27.2	2217	26.8
18–20	807	20.0	1224	25.5	1661	22.3	1801	21.7
21–24	1056	26.2	556	11.6	2394	32.1	2563	30.9
More than 24	535	13.3	1120	23.3	1367	18.4	1706	20.6
**Mother’s education**								
No education	3089	76.7	3811	66.6	4155	55.8	4178	50.4
Primary	372	9.2	789	13.8	1230	16.5	1101	13.3
Middle	–	–	–	–	587	7.9	–	–
Secondary	503	12.5	763	13.3	792	10.6	1747	21.1
higher	65	1.6	361	6.3	682	9.2	1261	15.2
**Mother’s occupation**								
Not working	3422	85.0	4092	71.5	5378	72.2	7183	86.7
Unskilled	64	1.6	158	2.8	938	12.6	46	.6
Skilled	495	12.3	1364	23.8	990	13.3	806	9.7
Prof/tech/managerial	45	1.1	106	1.9	140	1.9	249	3.0
**Type of residence**								
Urban	2011	49.9	1998	34.9	2244	30.1	3738	45.1
Rural	2018	50.1	3726	65.1	5202	69.9	4549	54.9
**Region**								
Punjab	1355	33.6	2305	40.3	4180	56.1	1740	21.0
Sindh	1066	26.5	1626	28.4	1714	23.0	1474	17.8
KPK	1043	25.9	1113	19.4	1117	15.0	1386	16.7
Balochistan	565	14.0	680	11.9	348	4.7	1005	12.1
Gilgit Baltistan	–	–	–	–	56	0.7	614	7.4
Islamabad (CT)	–	–	–	–	31	0.4	546	6.6
AJK	–	–	–	–	–	–	870	10.5
FATA	–	–	–	–	–	–	652	7.9
**Wealth index**								
Poorest	–	–	1289	22.5	1698	22.8	1827	22.0
Poorer	–	–	1235	21.6	1544	20.7	1863	22.5
Middle	–	–	1118	19.5	1464	19.7	1622	19.6
Richer	–	–	1064	18.6	1469	19.4	1486	17.9
Richest	–	–	1018	17.8	1272	17.1	1489	18.0

**Table 2:** Socio-demographics and pregnancy-related variables associated with caesarean deliveries (weighted percentages)

	PDHS 1990–91 (***n*** = 4029)	PDHS 2006–07 (***n*** = 5721)	PDHS 2012–13 (***n*** = 7461)	PDHS 2017–18 (***n*** = 8287)
**Rate of caesarean deliveries**	3.2%	7.8%	13.6%	19.6%
**Socio-demographics**				
**Mother’s age at birth (in years)**				
Less than 18	2.4%	3.8%	7.2%	9.3%
18–20	2.5%	6.6%	11.9%	15.3%
21–24	4.5%	7.6%	18.9%	21.8%
More than 24	4.1%	9.3%	27.6%	34.3%
**Children’s birth order**				
1	6.3%	14.3%	25.5%	27.4%
2–3	3.6%	10.5%	19.6%	24.2%
4–5	3.2%	6.1%	10.5%	17.4%
6 and above	1.2%	2.9%	6.1%	7.0%
**Mother’s education**				
No education	1.4%	3.9%	7.5%	9.1%
Primary	5.1%	10.4%	17.1%	18.3%
Middle	–	–	21.3%	–
Secondary	10.8%	16.4%	31.5%	28.8%
Higher	16.9%	25.3%	40.2%	42.9%
**Mother’s occupation**				
Not working	3.3%	8.8%	18.1%	19.4%
Unskilled	1.6%	5.1%	11.4%	15.2%
Skilled	2.3%	4.0%	5.3%	16.0%
Professional/Technical/ Managerial	11.4%	21.9%	30.0%	39.5%
**Wealth index**				
Poorest	–	1.9%	7.5%	6.1%
Poorer	–	2.4%	17.1%	9.8%
Middle	–	5.6%	21.3%	18.6%
Richer	–	11.3%	31.5%	29.0%
Richest	–	20.6%	40.2%	40.2%
**Type of residence**				
Rural	1.2%	5.1%	11.5%	14.4%
Urban	5.2%	12.8%	25.6%	26.0%
**Region**				
Baluchistan	0.7%	2.2%	1.7%	5.7%
Islamabad Capital Territory	–	–	28.1%	31.7%
Punjab	4.8%	11.4%	19.1%	31.0%
Sindh	3.5%	7.4%	17.4%	27.0%
Khyber Pakhtunkhwa	2.1%	4.4%	5.2%	10.0%
Gilgit Baltistan	–	–	3.6%	12.1%
AJK	–	–	–	25.4%
FATA	–	–	–	3.8%
**Pregnancy-related variables**				
**Pregnancy complication**				
No	–	9.0%	14.9%	–
Yes	–	17.9%	25.2%	–
**Number of antenatal care visits**				
None	1.0%	4.0%	22.1%	1.9%
1–3	4.6%	11.3%	10.7%	10.9%
4 and above	8.5%	19.3%	30.1%	31.3%
**Place of delivery**				
Public	15.9%	21.1%	28.9%	22.5%
Private	14.8%	21.3%	31.2%	34.9%
**Size of the baby at birth**				
Below average	13.9%	6.8%	37.9%	20.9%
Normal	13.0%	8.3%	32.1%	19.4%
**Number of children**				
1–2	4.9%	12.7%	20.4%	26.3%
3–4	3.6%	8.0%	13.4%	20.5%
5 and above	1.7%	2.9%	5.8%	8.7%
**Pregnancy ever terminated**				
No	–	9.9%	13.2%	20.9%
Yes	–	8.5%	14.5%	21.9%

### Simple binary logistic regression

The analysis of data in simple binary logistic regression models (Table 3) revealed that the age of the mother was significantly associated with caesarean section deliveries in all datasets of PDHSs, with higher rates among women with higher ages. Furthermore, the children’s birth order was strongly associated with the caesarean section deliveries. This is also visible for the mother’s education, as higher education is associated with a higher likelihood of caesarean section deliveries. This association overall applies to the mother’s education and employment. The likelihood of caesarean deliveries among women living in urban compared to rural areas was inconsistent between the four PDHS waves.

**Table 3:** Simple binary logistic regression of socio-demographics and pregnancy-related variables associated with caesarean deliveries

	PDHS 1990–91 (***n*** = 4029)			PDHS 2006–07 (***n*** = 5721)			PDHS 2012–13 (***n*** = 7461)			PDHS 2017–18 (***n*** = 8287)		
	OR	95% CI	***p***-value	OR	95% CI	***p***-value	OR	95% CI	***p***-value	OR	95% CI	***p***-value
**Socio-demographics**												
**Mother’s age at birth (in years)**												
Less than 18	1			1			1			1		
18–20	1.07	0.62–1.84	0.822	1.80	1.30–2.50	< 0.001	1.66	1.31–2.10	< 0.001	1.77	1.46–2.14	< 0.001
21–24	1.96	1.26–3.02	< 0.001	2.08	1.40–3.08	< 0.001	2.74	2.23–3.37	< 0.001	2.72	2.29–3.23	< 0.001
More than 24	1.78	1.05–3.04	< 0.001	2.60	1.91–3.55	< 0.001	5.00	4.04–6.19	< 0.001	5.10	4.28–6.07	< 0.001
**Children birth order**												
1	5.38	2.94–9.83	< 0.001	0.46	0.30–0.71	< 0.001	5.06	3.95–6.48	< 0.001	5.00	3.97–6.29	< 0.001
2–3	3.04	1.68–5.51	< 0.001	2.58	1.83–3.66	< 0.001	3.43	2.71–4.33	< 0.001	4.24	3.41–5.27	< 0.001
4–5	2.68	1.44–4.98	< 0.001	1.82	1.31–2.54	< 0.001	1.56	1.19–2.04	< 0.001	2.80	2.18–3.60	< 0.001
More than 6	1			1			1			1		
**Mother’s education**												
No education	1			1			1			1		
Primary	3.78	2.18–6.56	< 0.001	2.85	2.15–3.77	< 0.001	2.66	2.15–3.29	< 0.001	2.23	1.85–2.69	< 0.001
Middle	–	–	–	–	–	–	3.52	2.74–4.52	< 0.001	–	–	–
Secondary	8.46	5.60–12.78	< 0.001	4.81	3.74–6.19	< 0.001	5.22	4.26–6.40	< 0.001	4.05	3.49–4.69	< 0.001
Higher	14.19	6.94–29.01	< 0.001	8.31	6.23–11.09	< 0.001	7.86	6.51–9.49	< 0.001	7.51	6.45–8.76	< 0.001
**Mother’s occupation**												
Not working	1			1			1			1		
Unskilled	0.47	0.07–3.43	0.457	0.55	0.27–1.14	< 0.001	0.76	0.60–0.96	< 0.001	0.75	0.33–1.67	0.479
Skilled	0.69	0.37–1.29	0.241	0.44	0.03–0.59	< 0.001	0.36	0.25–0.51	< 0.001	0.79	0.65–0.97	< 0.001
Professional/Technical/Managerial	3.81	1.47–9.84	< 0.001	2.92	1.81–4.69	< 0.001	2.15	1.53–3.02	< 0.001	2.72	2.10–3.53	< 0.001
**Wealth index**												
Poorest	–	–	–	1			1			1		
Poorer	–	–	–	1.22	0.71–2.09	0.475	2.66	2.15–3.29	< 0.001	1.67	1.31–2.13	< 0.001
Middle	–	–	–	3.02	1.89–4.84	< 0.001	3.52	2.74–4.52	< 0.001	3.51	2.79–4.41	< 0.001
Richer	–	–	–	6.43	4.14–9.97	< 0.001	5.22	4.26–6.40	< 0.001	6.25	5.01–7.80	< 0.001
Richest	–	–	–	13.14	8.60–20.08	< 0.001	7.86	6.51–9.49	< 0.001	10.30	8.29–12.80	< 0.001
**Type of residence**												
Rural	1			1			1			1		
Urban	4.50	2.88–7.06	< 0.001	0.37	0.30–0.45	< 0.001	2.43	2.12–2.78	< 0.001	0.48	0.43–0.54	< 0.001
**Region**												
Baluchistan	1			1			1			1		
Islamabad Capital Territory	–	–	–				0.06	0.04–0.10	< 0.001	0.31	0.23–0.40	< 0.001
Punjab	0.15	0.05–0.41	< 0.001	0.62	0.49–0.78	< 0.001	0.71	0.56–0.89	< 0.001	0.82	0.71–0.96	< 0.001
Sindh	0.73	0.49–1.11	0.140	0.36	0.26–0.49	< 0.001	0.66	0.52–0.83	< 0.001	0.25	0.20–0.30	< 0.001
Khyber Pakhtunkhwa	0.43	0.23–0.71	0.001	0.18	0.10–0.30	< 0.001	0.20	0.15–0.27	< 0.001	0.14	0.10–0.18	< 0.001
Gilgit Baltistan	–	–	–	–	–	–	0.16	0.11–0.23	< 0.001	1.03	0.84–1.27	0.761
AJK	–	–	–	–	–	–	–	–	–	0.76	0.63–0.91	< 0.001
FATA	–	–	–	–	–	–	–	–	–	0.09	0.06–0.13	< 0.001
**Pregnancy-related variables**												
**Pregnancy complication**												
No	–	–	–	1			1			–	–	–
Yes	–	–	–	2.20	1.78–2.72	< 0.001	1.68	1.45–1.94	< 0.001	–	–	–
**Number of antenatal care visits**												
None	1			1			1			1		
1–3	5.08	2.97–9.68	< 0.001	3.05	2.17–4.28	< 0.001	6.21	4.15–9.31	< 0.001	6.31	4.15–9.61	< 0.001
4 and above	9.70	6.01–15.72	< 0.001	5.76	4.63–7.15	< 0.001	25.40	17.22–37.47	< 0.001	23.36	15.52–35.18	< 0.001
**Place of delivery**												
Public	1			1			1			1		
Private	0.92	0.61–1.39	0.696	1.01	0.81–1.26	0.938	1.33	1.14–1.54	< 0.001	1.85	1.64–2.09	< 0.001
**Size of the baby at birth**												
Below average	1			1			1			1		
Normal	1.08	0.38–3.07	0.888	0.81	0.63–0.99	< 0.001	1.29	0.95–1.75	0.103	1.10	1.26	0.180
**Number of children**												
1–2	0.34	0.21–0.53	< 0.001	0.21	0.15–0.27	< 0.001	0.24	0.20–0.29	< 0.001	0.27	0.23–0.32	< 0.001
3–4	0.72	0.48–1.09	0.116	0.60	0.48–0.75	< 0.001	0.60	0.52–0.70	< 0.001	0.72	0.64–0.82	< 0.001
5 and above	1			1			1			1		
**Pregnancy ever terminated**												
No	–	–	–	1			1			1		
Yes	–	–	–	1.12	0.90–1.40	0.310	1.11	0.97–1.28	0.136	1.06	0.80–1.41	0.675

Mothers having had pregnancy complications previously were significantly more likely to deliver via caesarean section in 2006–07 (OR = 2.20, 95% CI: 1.78–2.72) and 2012–13 (OR = 1.68, 95% CI: 1.45–1.94), whereas data were not available for 1990–91 and 2017–18. Similarly, the trend of caesarean deliveries among mothers who received antenatal care four times and more remained significant in all four datasets. Additionally, a significant relationship was found among mothers delivering babies in private sector hospitals and caesarean deliveries in 2012–13 (OR = 1.33, 95% CI: 1.14–1.54) and 2017–18 (OR = 1.85, 95% CI: 1.64–2.09). Moreover, no significant association was found between the size of the baby at birth and caesarean deliveries in three of the datasets of PDHSs (1990–91, 2012–13 and 2017–18). However, in 2006–07, mothers having a normal size baby at birth (OR = 0.81, 95% CI: 0.63–0.99) had higher chances of caesarean deliveries. Pregnancy termination was insignificant in all three datasets in which data was available. Furthermore, data showed that mothers having 1–2 children were significantly related with caesarean deliveries in all four datasets.

### Multivariable logistic regression

Using multivariable logistic regression analysis (Table 4), the data showed that mothers who had antenatal care visits more than four times had a higher likelihood of delivering babies through caesarean section in all four datasets. Pregnancy complications were also significantly associated in the two waves where the item has been assessed. Mothers aged more than 24 years were found to have a greater chance of giving birth through caesarean section in 2012–13 (AOR = 1.73, 95% CI: 1.37–2.24) and 2017–18 (OR = 1.79, 95% CI: 1.43–2.22). Mothers with a first child remained significant only in the PDHS 2017–18 (AOR = 0.13, 95% CI: 0.07–0.22). Moreover, mothers with higher education were found to be associated with caesarean section deliveries in 1990–91 (AOR = 8.60, 95% CI: 2.00–36.92) and 2017–18 (AOR = 1.35, 95% CI: 1.07–1.69).

**Table 4:** Multivariable logistic regression of socio-demographics and pregnancy-related variables associated with caesarean deliveries

	PDHS 1990–91 (***n*** = 4029)			PDHS 2006–07 (***n*** = 5721)			PDHS 2012–13 (***n*** = 7461)			PDHS 2017–18 (***n*** = 8287)		
	AOR	95% CI	***p***-value	AOR	95% CI	***p***-value	AOR	95% CI	***p***-value	AOR	95% CI	***p***-value
**Socio-demographics**												
**Mother’s age at birth (in years)**												
Less than 18	–	–	–	1			1					
18–20	–	–	–	1.26	0.85–1.85	0.250	1.10	0.84–1.44	0.511	1.12	0.90–1.41	0.315
21–24	–	–	–	1.31	0.81–2.22	0.267	1.40	1.09–1.78	< 0.001	1.30	1.06–1.60	< 0.001
More than 24	–	–	–	1.30	0.89–1.92	0.179	1.73	1.37–2.24	< 0.001	1.79	1.43–2.22	< 0.001
**Children’s birth order**												
1	5.99	0.89–40.18	0.650	0.90	0.46–1.77	0.758	1.17	0.67–2.04	0.590	0.13	0.07–0.22	< 0.001
2–3	7.30	1.48–36.02	0.151	2.04	1.09–3.80	< 0.001	0.90	0.54–1.50	0.676	0.13	0.07–0.23	< 0.001
4–5	8.10	1.54–37.04	0.104	1.63	1.00–2.66	< 0.001	0.70	0.46–1.06	0.090	0.11	0.06–0.20	< 0.001
6 and above	1			1			1			1		
**Mother’s education**												
No education	1			1			1			1		
Primary	1.91	0.59–6.15	0.279	1.28	0.86–1.91	0.219	1.04	0.80–1.35	0.788	0.86	0.68–1.08	0.182
Middle	–	–	–	–	–	–	–	–	–	–	–	–
Secondary	4.93	1.97–12.34	< 0.001	1.21	0.81–1.80	0.353	1.04	0.81–1.34	0.740	1.09	0.89–1.34	0.387
Higher	8.60	2.00–36.92	< 0.001	0.99	0.56–1.73	0.985	1.12	0.84–1.48	0.440	1.35	1.07–1.69	< 0.001
**Mother’s occupation**												
Not working	–	–	–	1			1			1		
Unskilled	–	–	–	1.50	0.63–3.57	0.360	0.72	0.44–1.15	0.169	0.68	0.13–3.54	0.642
Skilled	–	–	–	0.89	0.58–1.36	0.583	1.06	0.80–1.42	0.671	1.09	0.74–1.62	0.659
Professional/Technical/Managerial	–	–	–	1.05	0.44–2.51	0.912	1.04	0.70–1.54	0.858	0.78	0.43–1.41	0.407
**Wealth index**												
Poorest	–	–	–	1			1			1		
Poorer	–	–	–	0.82	0.38–1.77	0.614	1.01	0.67–1.50	0.979	1.33	0.99–1.78	0.058
Middle	–	–	–	1.72	0.87–3.41	0.122	1.06	0.72–1.57	0.770	1.66	1.24–2.23	< 0.001
Richer	–	–	–	2.57	1.30–5.07	< 0.001	1.38	0.93–2.05	0.115	1.98	1.46–2.68	< 0.001
Richest	–	–	–	3.25	1.57–6.70	< 0.001	1.66	1.08–2.54	< 0.001	2.20	1.59–3.05	< 0.001
**Type of residence**												
Rural	1			1			1			1		
Urban	2.37	0.77–7.31	0.135	1.12	0.81–1.55	0.507	1.75	1.44–2.13	< 0.001	1.04	0.90–1.22	0.584
**Region**												
Baluchistan	1			1			1			1		
Islamabad Capital Territory	–	–	–	–	–	–	0.38	0.23–0.64	< 0.001	0.39	0.28–0.52	< 0.001
Punjab	0.30	0.07–1.38	0.122	0.69	0.49–0.96	< 0.001	1.95	1.48–2.57	< 0.001	0.88	0.73–1.07	0.209
Sindh	0.37	0.16–0.84	< 0.001	0.39	0.25–0.61	< 0.001	1.44	1.08–1.93	< 0.001	0.27	0.21–0.34	< 0.001
Khyber Pakhtunkhwa	0.32	0.11–0.98	< 0.001	0.39	0.18–0.82	< 0.001	0.66	0.47–0.91	< 0.001	0.30	0.22–0.43	< 0.001
Gilgit Baltistan	–	–	–	–	–	–	0.47	0.30–0.73	< 0.001	0.70	0.54–0.89	0.004
AJK	–	–	–	–	–	–	–	–	–	0.80	0.64–1.00	0.054
FATA	–	–	–	–	–	–	–	–	–	0.18	0.11–0.28	< 0.001
**Pregnancy-related variables**												
**Pregnancy complication**												
No	–	–	–	1			1			–	–	–
Yes	–	–	–	1.80	1.34–2.42	< 0.001	1.31	1.11–1.55	0.001	–	–	–
**Number of antenatal care visits**												
None	1			1			1			1		
1–3	6.95	1.82–26.54	< 0.001	1.30	0.82–2.06	0.260	2.51	1.49–4.52	< 0.001	1.49	0.93–2.37	0.097
4 and above	9.64	2.61–35.61	< 0.001	2.26	1.64–3.11	< 0.001	4.24	4.67–6.42	< 0.001	2.21	1.39–3.52	< 0.001
**Place of delivery**												
Public	–	–	–	–	–	–	1			1		
Private	–	–	–	–	–	–	1.05	0.88–1.25	0.627	1.45	1.26–1.68	< 0.001
**Size of the baby at birth**												
Below average	–	–	–	1			–	–	–	1		
Normal	–	–	–	0.84	0.61–1.14	0.259	–	–	–	1.08	0.80–1.45	0.794
**Number of children**												
1–2	1.22	0.30–4.93	0.785	1.00	0.72–1.62	0.930	0.70	0.44–1.13	0.148	0.08	1.44–1.67	< 0.001
3–4	0.74	0.30–1.84	0.518	0.98	0.65–1.47	0.924	1.09	0.86–1.80	0.486	1.00	0.83–1.21	0.971
5 and above	1			1			1			1		
**Pregnancy ever terminated**												
No	–	–	–	–	–	–	–	–	–	1		
Yes	–	–	–	–	–	–	–	–	–	0.79	0.57–1.11	0.550

Multivariate logistic regression analysis was carried out to obtain AOR after entering all the independent variables simultaneously that were significant at level 0.05 in binary analysis

The mother’s occupation, pregnancy termination and size of the baby remained insignificant in all PDHS surveys. Mothers who belonged to the richest class showed a persistently strong association with caesarean deliveries in 2006–07 (AOR = 3.25, 95% CI: 1.57–6.70), 2012–13 (AOR = 1.66, 95% CI: 1.08–2.54) and 2017–18 (AOR = 2.20, 95% CI: 1.59–3.05). Considering the place of delivery, it was found that mothers who delivered babies in private hospitals were more likely to deliver children by caesarean compared to public hospitals (AOR = 1.45, 95% CI: 1.26–1.68) in PDHS 2017–18. Moreover, the results showed that mothers who had 1–2 children had higher chances of caesarean deliveries compared to 5 children and above only in 2017–18 (AOR = 0.08, 95% CI: 1.44–1.67).

## Discussion

The results reveal a drastic increase in caesarean deliveries among Pakistani women from 3.2% in 1990 to 19.6% in 2018. Further studies conducted in Pakistan reveal that foetal distress, prolonged labour pain, wound sepsis, previous caesarean history and rupture of placenta are the most common medical factors of caesarean section deliveries in the hospitals in Pakistan [16, 17]. The high cost of caesarean delivery is sometimes considered as a higher social status symbol. Such perception might be adding to the increasing trend of caesarean deliveries. Nevertheless, in Pakistan and other developing countries, where patients are rarely given an option to choose the mode of delivery, the doctor’s referrals for caesarean delivery can be a possible reason behind the increasing trend of caesarean deliveries [18].

The age of mothers at the time of delivery emerged as one of the contributing factors, as there is a strong relationship between the age of the mother and the mode of delivery (caesarean or vaginal). Many studies revealed that older mothers have a higher likelihood of caesarean deliveries [19, 20]. No research has been conducted to show the factors for the increased number of caesarean deliveries in the older mothers. However, it can be assumed that older mothers have more chances of having pregnancy complications that may result in caesarean deliveries [21]. Even in the absence of pregnancy-related complications, older mothers are more inclined to give birth through caesarean section [20]. Women in Pakistan have become more career conscious in terms of acquiring education and choosing a suitable job to earn their livelihood during the last few years [4]. The extensive process of becoming financially independent results into delayed marriages of women and, thus, increases their age for conception [22]. The increased age of a woman at the time of conception tends to have complications in the later period of the pregnancy that can possibly lead to a caesarean delivery [23]. Thus, it could be a possible reason for increased caesarean compared to vaginal deliveries.

Previous studies have revealed that women who reported having medical problems, such as chronic hypertension, chronic infections, heart problems or respiratory diseases, have a higher tendency of caesarean section births [24]. In correspondence to previous studies [24, 25], our results also showed that mothers who reported having complications, such as urinary tract infections, obesity or preeclampsia at any stage of pregnancy, had a higher tendency of caesarean delivery. On the other hand, PDHSs 1990–91 and 2017–18 did not include the questions related to pregnancy complications.

As per findings of previous clinical studies [26, 27], frequent visits to antenatal care facilities, pregnancy complications and the mode of delivering a baby are strongly associated. Considering the importance of antenatal care in the reduction of complications, a new World Health Organization guideline emphasises that every pregnant woman should have at least eight antenatal care visits during each pregnancy [4]. Findings of previous studies also revealed that antenatal care is considered an important component of women’s health during the course of pregnancy that can play a pivotal role in reducing the likelihood of caesarean deliveries [26–28]. By contrast, our study reveals a persistent upsurge of caesarean deliveries for mothers with more than four antenatal care visits more. Although there is no clear reason behind this, it can be assumed that women with pregnancy complications, such as obesity, hypertension and diabetes, were asked by the gynaecologists to have frequent antenatal care visits in order to handle any undesirable obstetric risk. While considering the regional demographics, the women from Punjab’s region persistently show a higher tendency towards caesarean deliveries. Feasible access and the availability of health care facilities at private and public hospitals can be traced as one of the factors behind increased caesarean deliveries in the province [4, 28].

Studies conducted in Bangladesh [29] and India [30] indicated that mothers belonging to a higher socio-economic class had a higher tendency to opt for caesarean section. In such cases, caesarean delivery seems to be a desire of woman who can afford it rather than the doctor’s referral for a safe childbirth process [31]. Moreover, research conducted in different countries also highlights that caesarean deliveries are more prevalent in women belonging to a higher socio-economic class as they have a misconception that caesarean delivery is the highest quality of obstetric care [6, 32]. Similarly, our study shows that a better socio-economic status is one of the significant factors behind increased caesarean section rates. It is obvious that a higher socio-economic status provides sufficient finances to bear the higher expenditure of caesarean surgery. Thus, caesarean section is more prevalent in women belonging to a higher socio-economic class [33]. On the other hand, women belonging to a lower socioeconomic status might not be able to afford the expenses of caesarean delivery or do not have access to the obstetric care facilities required for caesarean delivery. Therefore, the rate of caesarean deliveries is found to be lower in women having a poor socio-economic status [31].

Although it has been recognised that caesarean deliveries may support a reduction in the mother and child mortality rate, malpractices linked with caesarean surgery cannot be neglected [34]. A study conducted in India shows that the number of caesarean deliveries in private hospitals are three times higher than in public hospitals [33]. Similarly, the present study reveals that the rate of caesarean deliveries is increasing in private health care centres and hospitals in Pakistan over time. Arguably, the misuse of the surgical incision in private hospitals may involve varied factors. The high expenditure of a caesarean delivery, for example, can be considered as a significant factor that has resulted in increased rates of caesarean deliveries in private hospitals. Considering the situation in private hospitals, the doctors may prefer caesarean delivery over vaginal delivery for financial benefits and time convenience [33]. Additionally, the higher rates of caesarean deliveries in private hospitals tend not only to increase the cost of health care but they increase the health risks of women and newly born babies. However, the PDHSs (1990–2018) for both private and public hospitals lack the relevant information regarding the medical reasons for performing caesarean deliveries.

The literature shows that the low weight of baby at birth or a small size is not strongly associated with the caesarean delivery, as the mothers who were told about the low birth weight or the small size of the newborn at the time of birth had fewer chances to undergo caesarean delivery [33]. Similarly, results of the present study show that the size of the baby is not interlinked with the caesarean delivery, as mothers whose babies had below average size did not have a higher tendency to have a caesarean delivery.

### Limitations

The data from the PDHSs have some limitations that tend to affect the findings of the study. First of all, the cross-sectional design does not allow for causal interpretations. While taking into consideration pregnancy-related variables, the information related to pregnancy termination was not available in 2006–07 and 2012–13. Similarly, PDHS 2017–18 lacks the relevant information about pregnancy complications and, thus, affected the trend analysis of the respective variable in the present study. In addition, the information about birth weight is uncertain. Consequently, we used a combined variable on size and weight at birth to borrow strength. The data in all four PDHSs waves (1990–2018) do not offer relevant information on the nature of pregnancy complications experienced by a mother. Keeping in view the limited availability of data required, the present study is unable to highlight any particular pregnancy complication that led to a caesarean section delivery. The PDHS 1990–2018 data lacked the information to differentiate between medical and non-medical reasons for carrying out caesarean surgery, thus, the study could not discuss the misuse of surgical incision for child delivery as a contributing factor for the increase in the caesarean deliveries. Additionally, the dataset of PDHS 2017–18 does not include the information related to pregnancy complications.

## Conclusions

The findings of the present study reveal a progressive increasing trend of caesarean deliveries in Pakistan over time (from 3.2% in 1990 to 19.6% in 2018). The data revealed a strong relationship between independent variables, including the age of a mother at birth, mothers living in an urban area, residing in Punjab province, having a higher socio-economic status and using more antenatal care, and delivering in private hospitals and the occurrence of caesarean surgery as a delivery mode. The findings of the study further recommend that the health system in Pakistan should provide clear medical guidelines to doctors for carrying out caesarean section deliveries. Strict adherence to those medical guidelines will help in reducing the rate of caesarean sections in both public and private hospitals in Pakistan. Moreover, awareness programmes as a part of antenatal care can create awareness among women related to pregnancy complications and childbirth processes that tend to minimize the use of not medically caesarean deliveries which are not medically indicated.

## Data Availability

Data are available from the DHS programme at https://dhsprogram.com/.

## Abbreviations

AOR: Adjusted odds ratio
CI: Confidence interval
DHS: Demographic and Health Survey
OR: Odds ratio
PDHS: Pakistan Demographic and Health Survey
WHO: World Health Organization
